# Congenital Toxoplasmosis in France and the United States: One Parasite, Two Diverging Approaches

**DOI:** 10.1371/journal.pntd.0005222

**Published:** 2017-02-16

**Authors:** Francois Peyron, Rima Mc Leod, Daniel Ajzenberg, Despina Contopoulos-Ioannidis, François Kieffer, Laurent Mandelbrot, L. David Sibley, Hervé Pelloux, Isabelle Villena, Martine Wallon, Jose G. Montoya

**Affiliations:** 1 Hospices Civils de Lyon, Institut de Parasitologie et mycologie médicale, Hôpital de la Croix Rousse, Lyon, France; 2 Department of Ophthalmology and Visual Science, Department of Pediatrics (Infectious Diseases), The University of Chicago, Chicago, Illinois, United States of America; 3 Toxoplasmosis Center, Institute for Genomics and Systems Biology, The University of Chicago, Chicago, Illinois, United States of America; 4 Global Health Center, CHeSS, The University of Chicago, Chicago, Illinois, United States of America; 5 Centre National de Référence (CNR) Toxoplasmose / *Toxoplasma* Biological Resource Center (BRC), Centre Hospitalier-Universitaire Dupuytren, Limoges, France; 6 INSERM UMR 1094, Neuroépidémiologie Tropicale, Laboratoire de Parasitologie-Mycologie, Faculté de Médecine, Université de Limoges, Limoges, France; 7 Department of Pediatrics, Division of Infectious Diseases, Stanford University School of Medicine, Stanford, California, United States of America; 8 Neonatal Intensive Care Unit, Armand Trousseau Hospital–Assistance Publique–Hopitaux de Paris, Paris, France; 9 Hopital Louis Mourier, Assistance Publique–Hopitaux de Paris, Colombes, France; 10 Centre de Recherche en Epidémiologie et Sante des Populations, INSERM U1018, Le Kremlin-Bicêtre, France, Université Paris Diderot—Paris 7, Paris, France; 11 Department of Molecular Microbiology, Washington University School of Medicine, St. Louis, Missouri, United States of America; 12 Parasitologie-Mycologie Pole Biologie, CHU A. Michallon, Grenoble, France; 13 UMR 5163 CNRS/Université Grenoble Alpes, Grenoble, France; 14 Laboratoire de Parasitologie-Mycologie, Centre National de Référence de la Toxoplasmose, Centre de Ressources Biologiques Toxoplasma, Hôpital Maison Blanche Reims, France; 15 EA 3800, SFR CAP-SANTE, Université Champagne-Ardenne, Reims, France; 16 Physiologie intégrée du système d'éveil Centre de recherche en neurosciences de Lyon, INSERM U1028-CNRS UMR 5292, Lyon, France; 17 Department of Medicine and Division of Infectious Diseases, Stanford University School of Medicine, Stanford, California, United States of America; 18 Palo Alto Medical Foundation Toxoplasma Serology Laboratory, Palo Alto, California, United States of America; University of Zurich, SWITZERLAND

## Introduction

At 29 weeks of gestation, a pregnant woman in the United States was told that her fetal ultrasound had revealed the presence of hydrocephalus. She did not recall having any symptoms or risk factors during gestation. Serological testing at the national reference laboratory for toxoplasmosis in the US (http://www.pamf.org/serology/) confirmed an acute infection likely acquired around 17 weeks of gestation. She was started on pyrimethamine/sulfadiazine, at 31 weeks. Her amniotic fluid was positive for *Toxoplasma gondii* DNA by polymerase chain reaction (PCR). The infant was born with hydrocephalus, brain calcifications, and chorioretinitis. In France, a screening program has been in place since 1992, and pregnant women with negative serology are tested monthly until delivery. If this pregnant woman had been followed according to this program, she would have been started on spiramycin 14 weeks earlier than in the US, her amniotic fluid would have been tested for *T*. *gondii* by PCR at 18 weeks, and she would have been started on pyrimethamine/sulfadiazine 11 to 8 weeks earlier than in the US. In the US, such a program does not exist, and infection is usually diagnosed when clinical signs are present in the fetus or at birth.

Severe disease is not an uncommon situation in the US [1], but it is apparently a rare event in France (Table 1) [2]. Why is a congenital infection caused by the same parasite approached so differently in France than in the US? Below, we will discuss several factors that could account for these diverging approaches.

**Table 1:** Differences in *T*. *gondii* genetics, epidemiology, clinical manifestations, and approach to infection detection and management during pregnancy, between France and the US.

	France	US
**Parasite Genetics**	Type II: >95%; other types are very rare	Type II: 41.5%; non-type II (including atypicals): 58.5%
**IgG Seroprevalence in Women of Childbearing Age**	37.0%	9.1%
**Incidence of Acute Infection among *Toxoplasma*-Seronegative Pregnant Women**	2.1/1,000	0.2/1,000*		
**Incidence of Congenital Toxoplasmosis**	2.9/10,000 live births	0.5/10,000 live births*		
**Clinical Signs of Congenital Toxoplasmosis in Newborns: Absent, Mild–Moderate, and Severe**	85%, 10%, and 3%	12%, 11%, and 77%		
**Screening of *Toxoplasma*-Seronegative Pregnant Women**	Yes. Systematic screening is performed every month	No. However, systematic screening is performed in some obstetric practices		
**Diagnosis of Acute *T*. *gondii* Infection by Seroconversion**	Yes, because sequential samples are available	Rare		
**Diagnosis of Acute *T*. *gondii* Infection by the Use of a Single Serum**	Rare	Yes. Only a single serum is available. Positive *Toxoplasma* IgM samples require confirmatory testing at reference centers such as the Palo Alto Medical Foundation Toxoplasma Serology Laboratory**		
**Recommendation of Treatment, Amniotic Fluid for *T*. *gondii* PCR Testing, and Serial Ultrasounds for Acutely Infected Women**	Yes	Yes		
**Indication of Infant's Workup for Congenital Toxoplasmosis at Birth**	Yes, for each newborn born to a mother infected during gestation regardless of the presence of clinical signs or laboratory/radiological abnormalities	Yes, for those with clinical signs and/or laboratory/radiological abnormalities suggestive of congenital infection. Seldom, for infected infants without clinical signs or laboratory/radiological abnormalities whose mothers were suspected of having or diagnosed with toxoplasmosis during gestation		
**Postnatal Treatment of Congenitally Infected Infants**	Yes, for infected infants of acutely infected pregnant women (symptomatic or asymptomatic) diagnosed with congenital toxoplasmosis in utero or postnatally, regardless of the presence of clinical, laboratory, or radiological abnormalities	Yes, for infants diagnosed with congenital toxoplasmosis because of the presence of clinical signs in utero or at birth. Seldom, for infected infants without clinical, laboratory, or radiological abnormalities in whom the diagnosis of congenital toxoplasmosis was made in utero or postnatally because of the presence of maternal illness, risk factors, or systematic screening by an obstetric practice		

Ig, immunoglobulin.

*These estimates are based on data from the New England Newborn Screening Program and not on data from active surveillance. In this program, the incidence of acute infection among *Toxoplasma*-seronegative pregnant women and the incidence of congenital infection are underestimated since fetal losses due to toxoplasmosis during gestation are not included, the filter paper used for screening is only 50% to 75% sensitive, detection of *Toxoplasma* IgA is not used, and the actual denominator of women at risk (those who are *Toxoplasma* seronegative at the beginning of pregnancy) is not known.

**http://www.pamf.org/serology/

## Parasite Genetics

Approximately 95% of *T*. *gondii* strains infecting humans in France are type II (Table 1) [3]. In contrast, a more heterogeneous distribution of strains is observed in the US. This may reflect greater migration from South America, where strain types are diverse, or from natural isolates in the wild, which are more heterogeneous. Sampling, mainly of animal isolates, in North America indicates that type II represents less than half of the *T*. *gondii* strains (43.9%) [4].

## Epidemiology

In France, toxoplasmosis seroprevalence has notably decreased in pregnant women, from 83% in 1965 to 37% in 2010 [5]. Estimation of the incidence of seroconversion during pregnancy in 2010 was 2.1 per 1,000 susceptible pregnant women [6]. The incidence rate of congenital toxoplasmosis in 2007 at birth was estimated at 2.9 (95% CI 2.5 to 3.2) per 10,000 live births (Table 1) [7]. In the US, the seroprevalence of toxoplasmosis among women of childbearing age (15–44 years) has also declined from 14.9% in a 1988–1994 survey to 9.1% in a 2009–2010 survey. The incidence of *T*. *gondii* infection during pregnancy at delivery and 6 weeks postpartum was estimated to be 1.1/1,000 pregnant women [8].The incidence of congenital toxoplasmosis, based on results from the New England Newborn Screening Program, was estimated to be 0.82 cases per 10,000 live births for the period of 1986–1992 [9]. It is currently estimated at 0.5 cases/10,000 live births (see Table 1).

## Screening Programs

In France, all susceptible (seronegative) pregnant women are tested monthly until delivery. The objective is to promptly identify and treat maternal infection in order to prevent fetal infection and to decrease sequelae in the infected offspring. In the US, pregnant women are rarely screened for toxoplasmosis. The vast majority of physicians in the US indicate recommend toxoplasmosis testing only for pregnant women who have an abnormal fetal ultrasound suggestive of congenital infection or, less commonly, have risk factors for acute infection or present with an illness suggestive of toxoplasmosis. This strategy has been shown to miss at least half of mothers who give birth to congenitally infected offspring [10]. Newborn screening for toxoplasmosis is only practiced in Massachusetts and New Hampshire [9].

## Treatment

In France, spiramycin is given to mothers as soon as infection is suspected. It is given continuously until delivery unless amniotic fluid PCR turns out to be positive, in which case it is switched to pyrimethamine/sulfadiazine or pyrimethamine/sulfadoxine. These drugs are given continuously until delivery. In the US, the approach to treating pregnant women diagnosed with maternal toxoplasmosis is similar: for infection acquired during the first 18 weeks of gestation or shortly before conception, spiramycin is recommended until delivery [11]. If fetal infection is confirmed by a positive result in PCR of amniotic fluid, treatment with pyrimethamine/sulfadiazine is recommended (Table 1). Termination of pregnancy (TOP) is an option in France for fetuses with severe manifestations but is rarely performed in France, primarily because of the progress in prenatal diagnosis and treatment. In the US, approximately 20% of pregnant women who were told that they had a positive *Toxoplasma* immunoglobulin (Ig) M chose TOP before any further confirmatory tests and antenatal diagnosis of fetal infection were attempted [12].

## Prognosis

Congenital toxoplasmosis as managed in France has been reported to have little effect on the quality of life and visual function of the affected adults [13]. In a cohort of more than 700 congenitally infected children, there is no apparent evidence of a higher rate of neuropsychiatric diseases. During a median follow-up of 10.5 years, 30% of infected children born to treated mothers developed at least one new ocular lesion [14]. In contrast, in the US, upon long-term follow-up, 72% of children born to untreated mothers developed a new ocular lesion [15]. Overall, outcomes appear to be better in the era of treatment, even if treatment is instituted postnatally, than in earlier decades when treatment had not been implemented at all in the US [1,16]

## Discussion

In the case we report here, had the pregnant woman undergone regular screening for toxoplasmosis, she would have been told 3 months earlier that she had become infected and would have been given pyrimethamine/sulfadiazine/folinic acid 13 weeks earlier than in the US. Would an earlier treatment have changed the outcome of the fetus? Literature from countries in Europe where the French or French-like approach is followed has steadily reported good outcomes in children infected with toxoplasmosis when treatment is introduced shortly after maternal infection [2]. During the same period, literature from the US, where antenatal programs are lacking, has been reporting infected children with more severe disease and poorer outcomes [1]. How can we explain such divergent approaches to the same disease? Several factors like parasite or host genetics and infecting forms could account for this difference. The presence of antenatal treatment in France and its absence in the US certainly play an important role. Recent evidence from Europe and France suggests that treatment of toxoplasmosis during pregnancy is associated with lower transmission rates and decreased sequelae [2]. A meta-analysis of individual patient data demonstrated that transmission rate was significantly reduced by half when treatment was introduced 3 weeks versus 8 weeks after the estimated date of maternal infection [11]. Data from the French Lyon cohort study reported that when monthly screening was implemented in 1992 and compared to screening before 1992, the risk of fetal infection fell significantly (for example, at 26 weeks of gestation, it fell from 59.6% to 46.6%; *p* = 0.038) [2]. The same study reported that when PCR on amniotic fluid was routinely introduced in 1995 (i.e., infected fetuses were treated earlier with pyrimethamine/sulfadiazine), the risk of developing clinical signs in children followed for 3 years dropped significantly (odds ratio [OR] 0.59; 95% CI 0.4 to 0.89; *p* = 0.012), and the odds of severe neurologic sequelae or death in infants with congenital toxoplasmosis was also significantly lower (OR 0.24; 95% CI 0.07–0.71) [2]. A short delay between maternal infection and treatment onset significantly reduced the risk of ocular lesion during a 2-year follow-up [17]. Another study demonstrated that antenatal treatment significantly reduced the occurrence of severe neurologic sequelae or death [11]. Prusa-Romana et al. recently reported that antenatal treatment reduces the risk of mother-to-child transmission when compared with those without treatment [18]. Thus, it is possible that the greater disease severity observed at birth in the US is in part due to the lack of antenatal screening and treatment in these children.

France and US stand as paradigms of the divergent approaches to diagnosing and treating congenital toxoplasmosis; in reality, they represent a lack of consensus.

Systematic screening programs for all pregnant women at risk are not widely implemented, despite having been shown to work in several countries. Here we propose that, whatever the approach chosen (French-like versus USA-like), there are three underlying issues that need to be addressed by each country or region: parents should be informed about the disease and how to prevent it; health care providers should have access to state-of-the art information and guidelines, and counseling should be available at reference centers; and studies should be performed to address the issue of whether antenatal screening programs are cost-effective. There are some concerns regarding the future financial viability of these programs in light of the decline in prevalence [5]. However, such programs could be cost-effective even in the US assuming maternal screening test costs are lower than those currently offered [19]. Efforts towards reducing the cost of laboratory testing should be a top priority.
